# Superficial ulnar artery pseudoaneurysm

**DOI:** 10.1590/1677-5449.202300471

**Published:** 2023-12-04

**Authors:** Mariana Jordão França, Luciana Akemi Takahashi, Graciliano José França, Claudio Augusto Carvalho, Maria Alice Zarate Nissel

**Affiliations:** 1 Universidade Positivo – UP, Curitiba, PR, Brasil.; 2 Universidade Federal do Paraná – UFPR, Hospital de Clínicas, Curitiba, PR, Brasil.

**Keywords:** ulnar artery, anatomic variant, pseudoaneurysm

## Abstract

The ulnar artery is the larger terminal branch of the brachial artery. It originates in the cubital fossa and is covered by the flexor muscles of the forearm. We report an anatomic variant in which the ulnar artery was in a superficial position in the forearm. Since this variant was unknown, an attempted venous puncture injured the artery, causing formation of a pseudoaneurysm.

## INTRODUCTION

Normally, the ulnar artery originates in the cubital fossa and is the larger of the two terminal branches that arise from division of the brachial artery. It courses distally and medially below the superficial flexor muscles of the forearm and is located over the deep flexor muscle of the fingers (flexor digitorum profundus). Within the distal two-thirds of the forearm, the ulnar artery is lateral to the ulnar nerve, in Guyon’s canal.^1^ Below the retinaculum of the flexors, it surfaces to form the superficial palmar arch, together with the superficial palmar branch of the radial artery.

In cases in which the ulnar artery follows a superficial path, it takes an unusual course, superficial to the flexor muscles of the forearm, and may originate either from the brachial artery or from the axillary artery. The prevalence of superficial ulnar artery is in the range of 0.7-9.4%,^2^ and it is more common unilaterally in the right arm. The prevalence of bilateral occurrence is 0.01-0.62%.^3^

Pseudoaneurysms of the peripheral arteries are rare, with much lower frequency in upper limbs than in lower limbs.^4^

The study protocol was approved by the Ethics Committee at our institution (decision number 5.702.203). A free and informed consent form for studies involving human beings was signed.

## PART I – CLINICAL SITUATION

**Figure 1:**
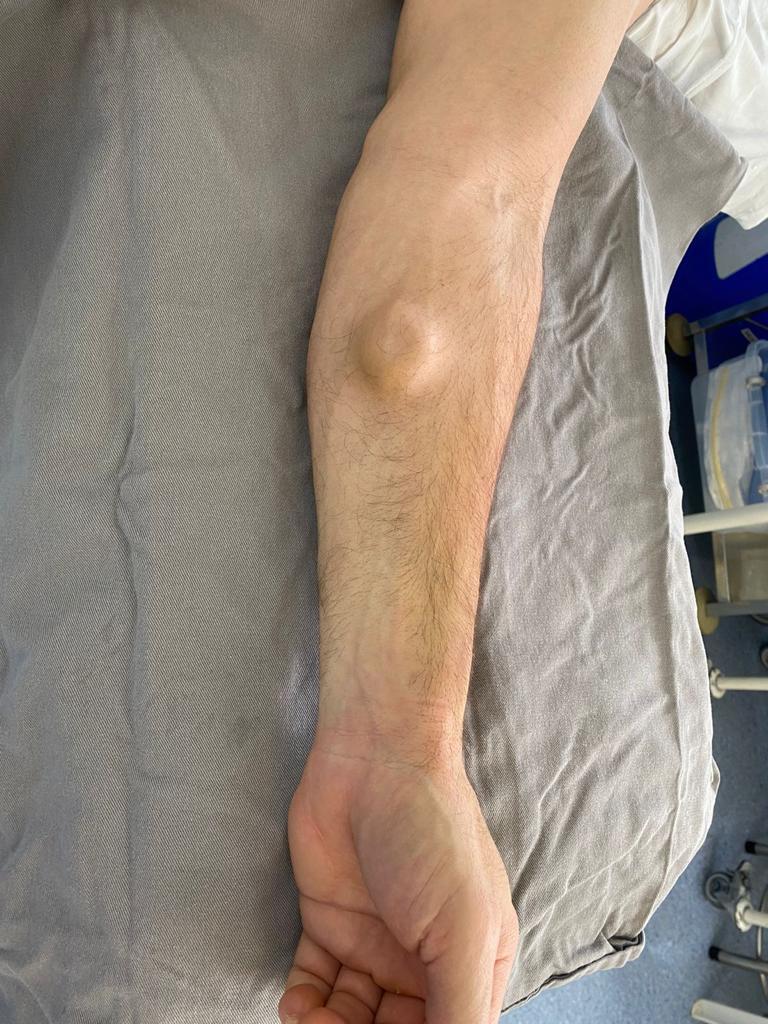
Pseudoaneurysm seen during physical examination.

**Figure 2:**
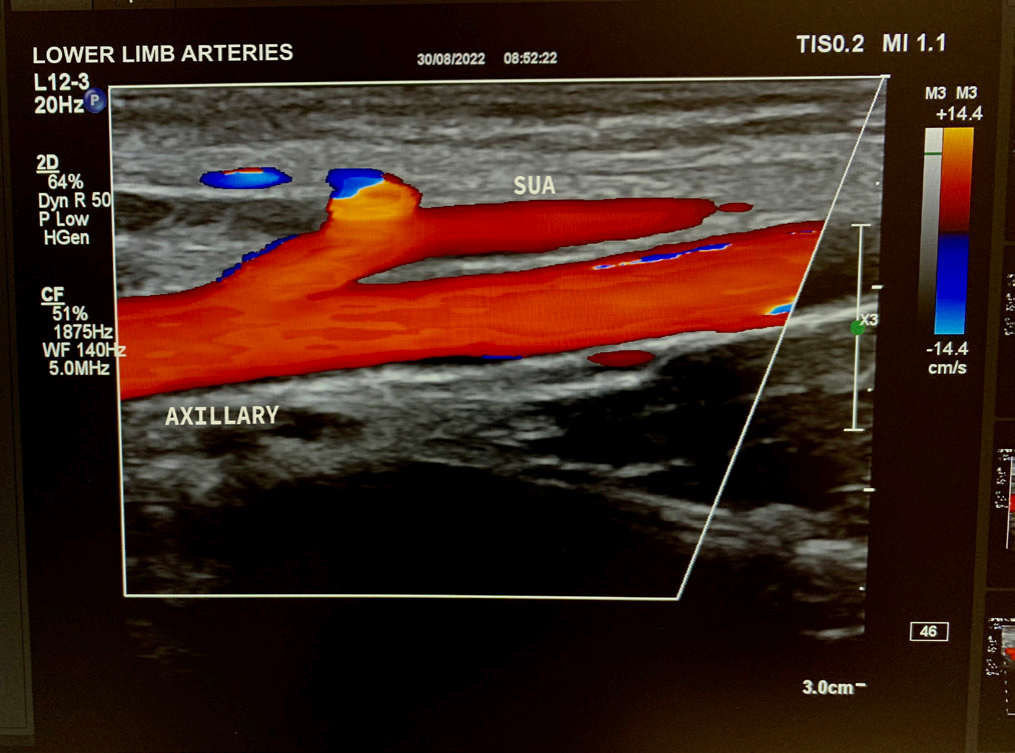
Superficial ulnar artery originating from the axillary artery.

**Figure 3:**
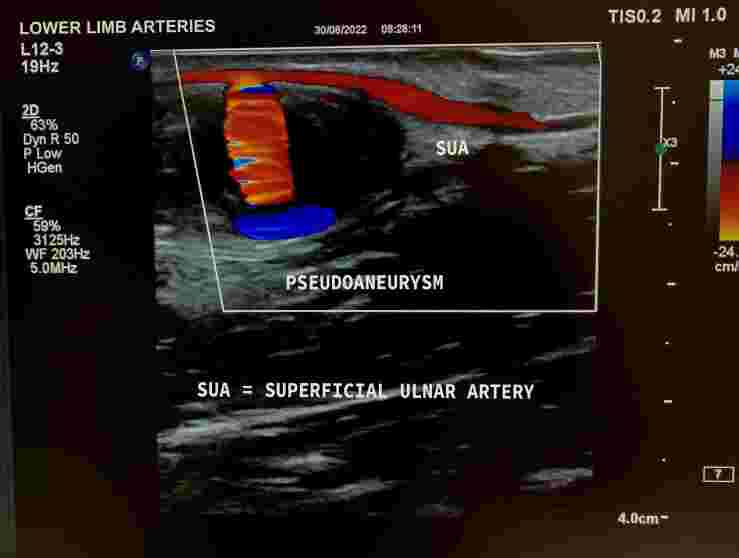
Doppler ultrasonography demonstrating the superficial ulnar artery and arterial flow into the pseudoaneurysm sac.

**Figure 4:**
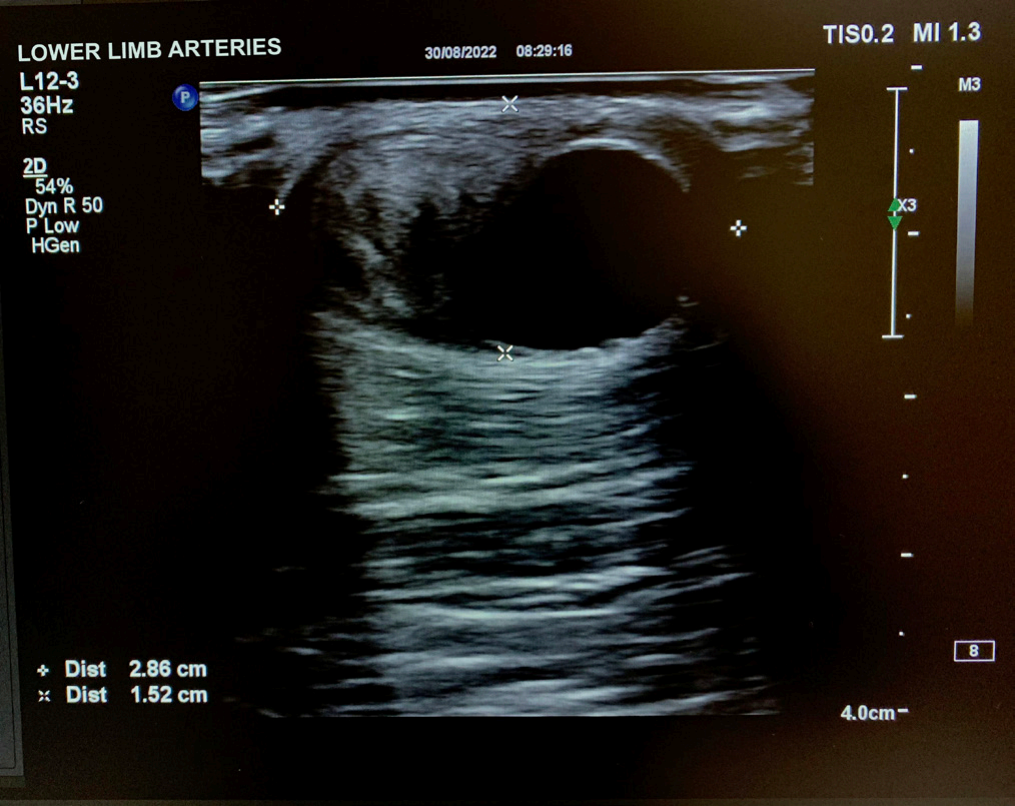
Measurement of the dimensions in B-mode.

**Figure 5:**
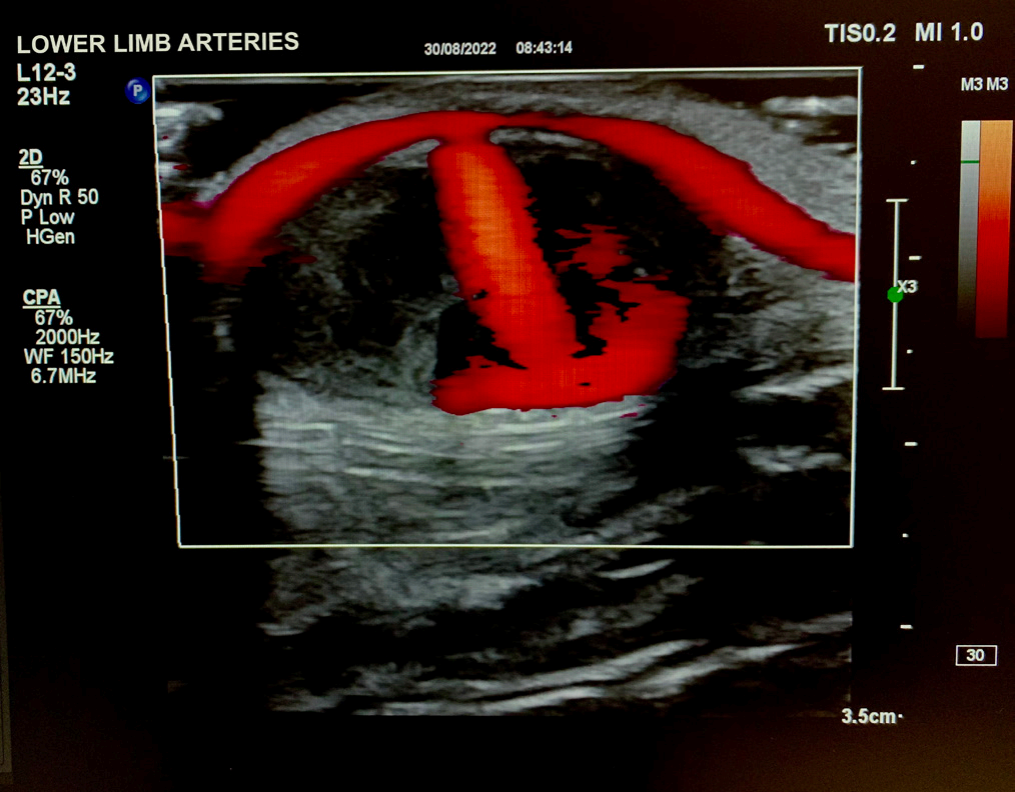
Doppler ultrasonography demonstrating the superficial ulnar artery and arterial flow into the pseudoaneurysm sac with Power Doppler.

The patient was a 53-year-old male mechanic with a history of hospital admission for coronary surgery. He presented at the cardiology clinic seeking medical care for a palpable and painless mass of approximately 4 cm in size that had formed on the anterior aspect of his forearm after an attempted venous puncture during the surgical procedure. After physical examination, color Doppler ultrasonography (CDU) of the arteries of the upper limbs was requested with the objective of identifying the nature of the palpable mass (Figure 1). Examination of the left upper limb revealed a patent superficial ulnar artery, originating from the axillary artery (Figure 2), free from stenosis, but with that had formed in its mid third. The pseudoaneurysm had mural thrombi and measured 2.86 by 1.52 cm (Figures 3, 4, and 5). There was no evidence of a similar anatomic variant in the right upper limb. During the CDU examination, it was concluded that the radial artery and the deep and superficial palmar arches were patent, ruling out the need for an Allen maneuver or any similar approach.

## PART II – WHAT WAS DONE

**Figure 6:**
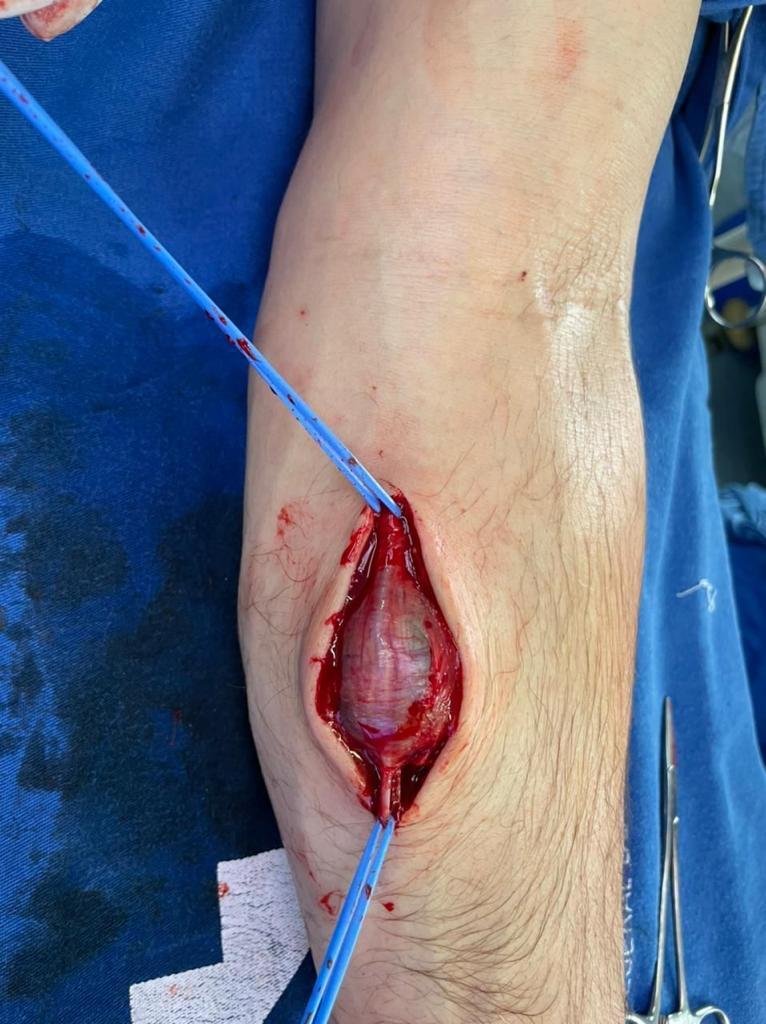
Superficial ulnar artery pseudoaneurysm after proximal and distal control.

**Figure 7:**
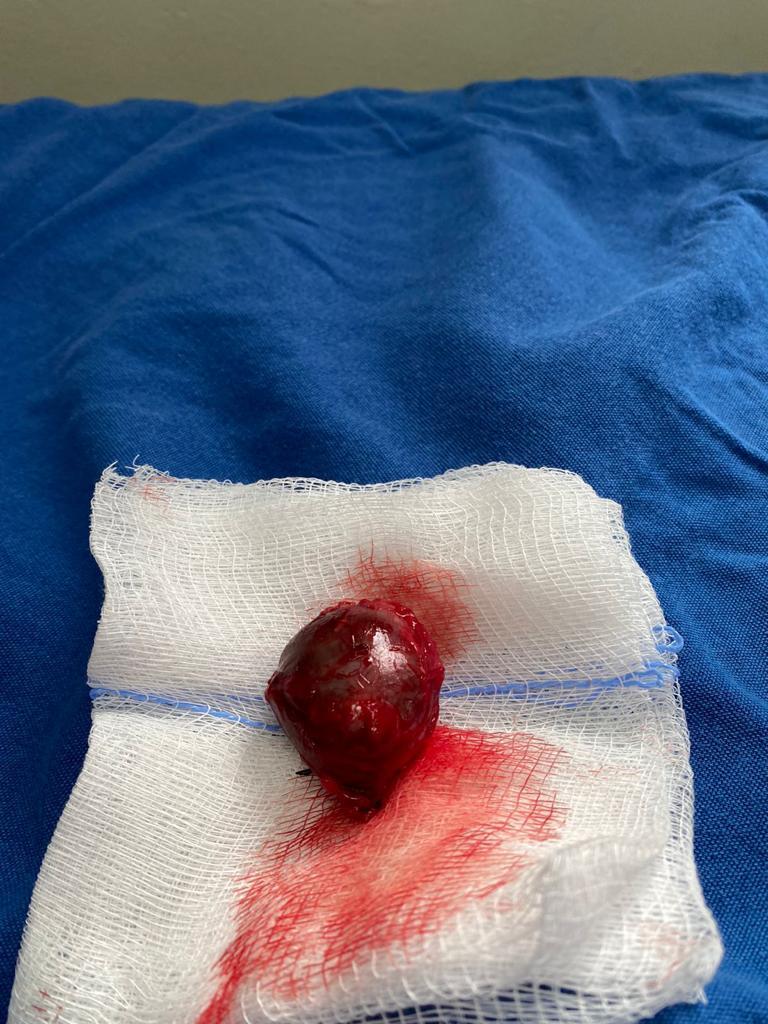
Pseudoaneurysm after resection.

**Figure 8:**
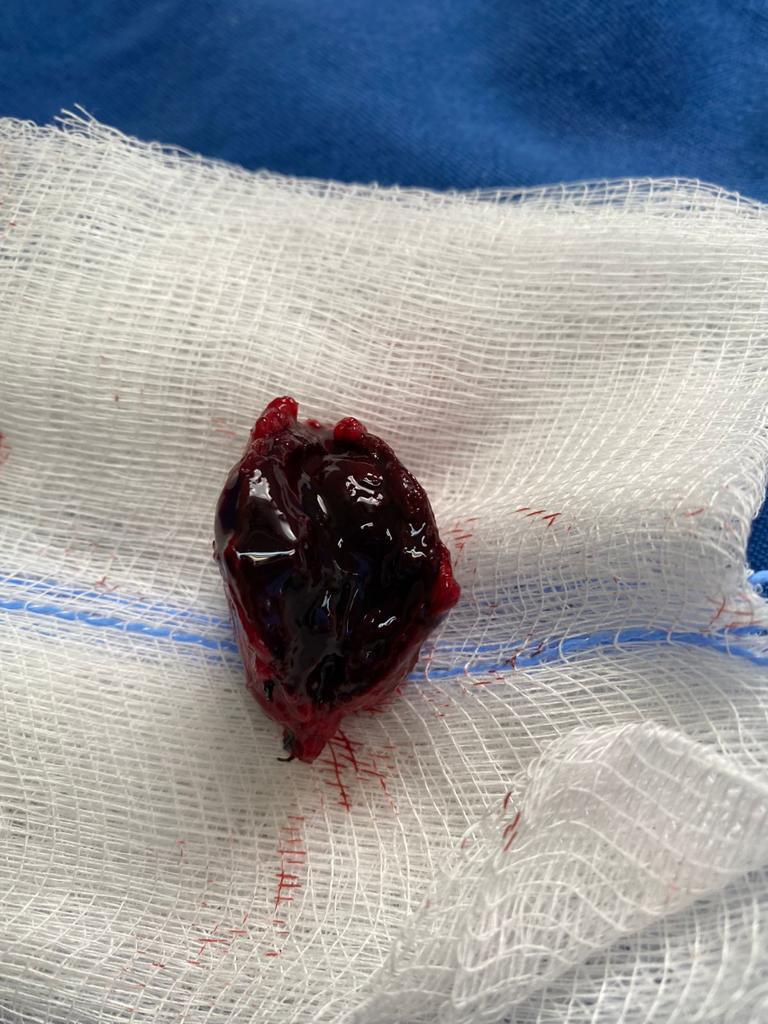
Pseudoaneurysm opened, showing recent thrombi.

**Figure 9:**
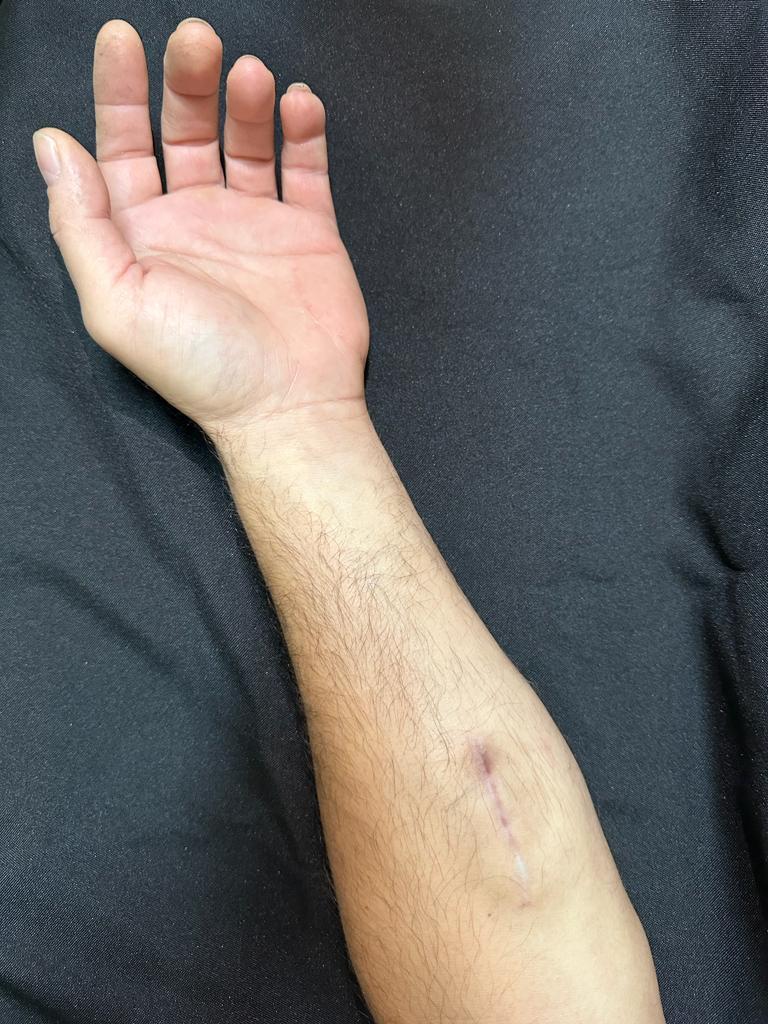
Appearance of surgical scar 3 months postoperatively.

**Figure 10:**
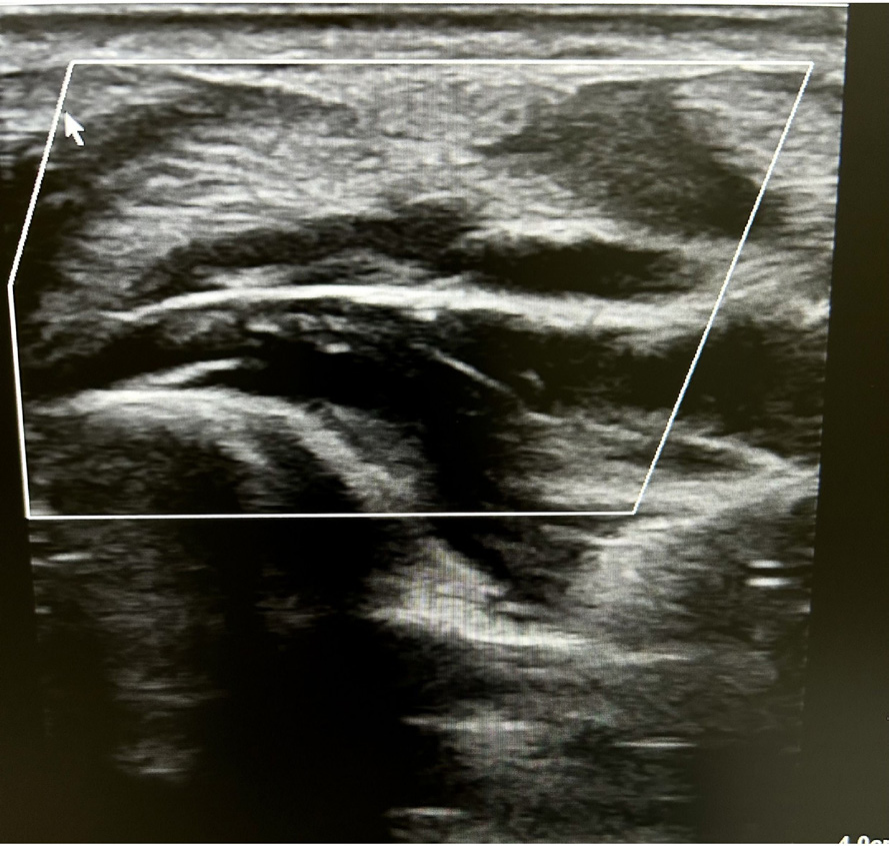
Absence of flow on Doppler along the path of the superficial ulnar artery.

**Figure 11:**
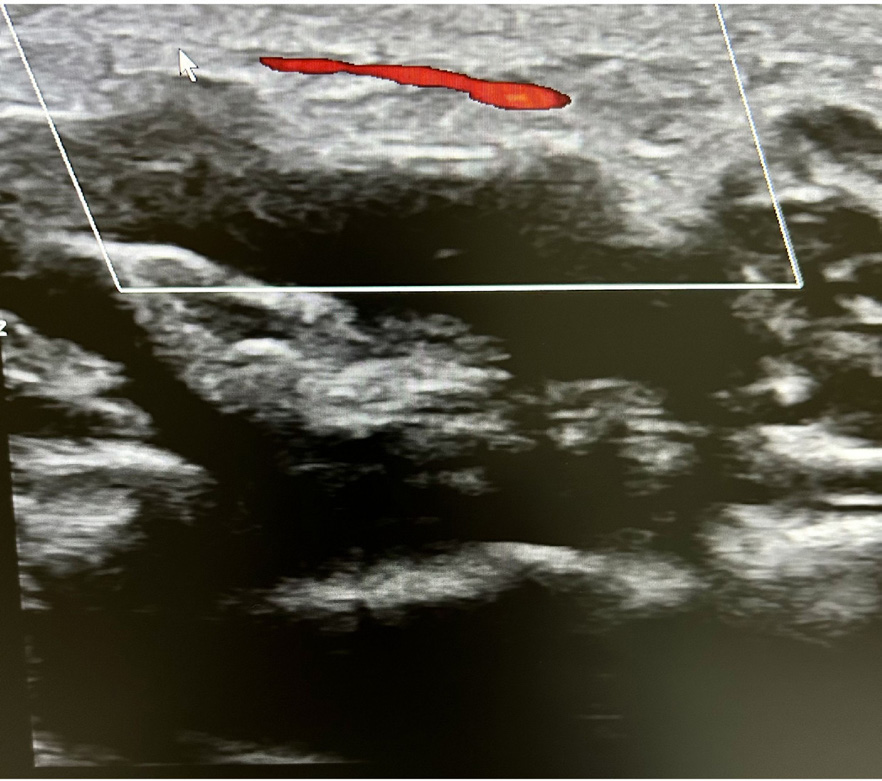
Superficial palmar arch patent on color Doppler ultrasonography.

**Figure 12:**
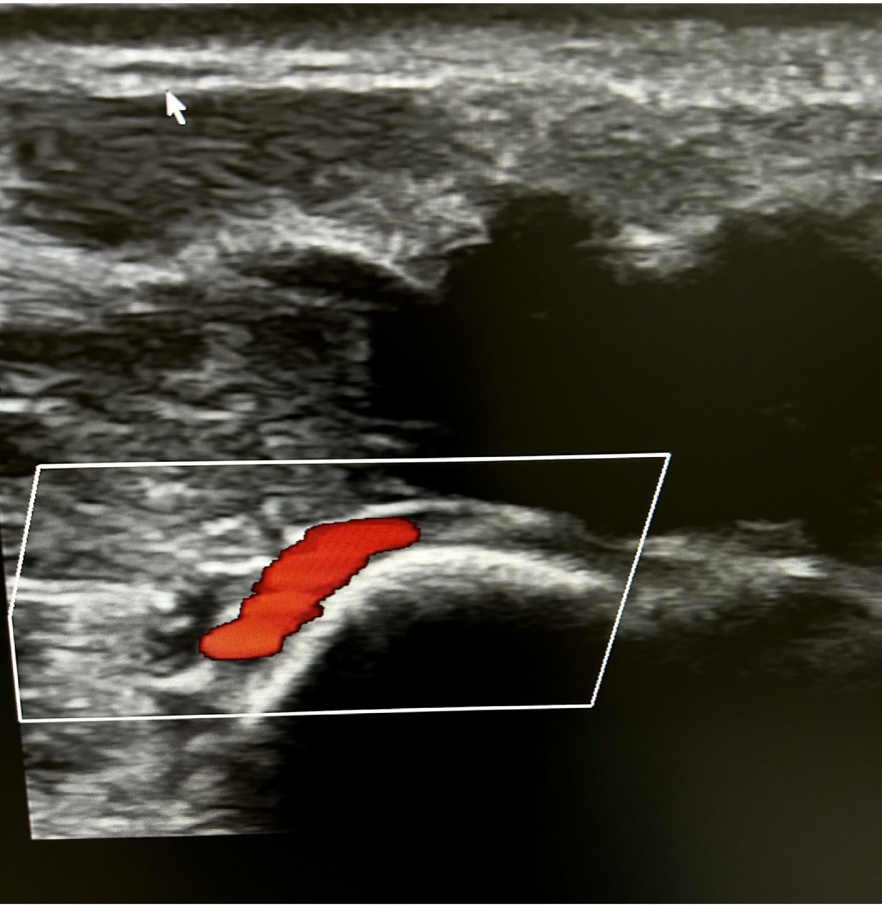
Deep palmar arch patent on color Doppler ultrasonography.

After the examination, a consultation was scheduled at the Vascular Surgery Clinic, where open surgery was chosen to treat the injury. The time elapsed between the examination and surgery was 50 days. The operation was initiated with a 4-5 cm incision over the pseudoaneurysm, with proximal and distal control of the superficial ulnar artery, in addition to separation of the two basilic veins (Figure 6). Next, dissection of the pseudoaneurysm was performed, ligating small collateral arteries. On opening the pseudoaneurysm, it was observed that there was total thrombosis of the aneurysm sac and of the distal ulnar artery (Figures 7 and 8). The surgical option chosen was therefore ligature of the proximal and distal ulnar artery, which showed no signs of reflux, and resection of the thrombosed pseudoaneurysm, followed by review of hemostasis and skin suture. There were no signs of ischemia of the hand during the immediate postoperative period and a control CDU showed that the deep and superficial palmar arches were patent. The patient returned to the Vascular Surgery Clinic 3 months after the operation (Figure 9), free from complaints, and was sent for a control CDU, which showed absence of flow through the superficial ulnar artery (Figure 10), absence of stenosis of the radial artery, and good flow through the palmar arches (Figures 11 and 12).

## DISCUSSION

The superficial ulnar artery anatomic variant is well described in the literature. In a study of 408 upper limbs, a superficial ulnar artery was found in 2.5% of cases.^5^ Another study, with 95 cadavers, reported a 4.2% prevalence of superficial ulnar artery, 75% of which were in the right upper limb.^6^ Other authors have identified incidence of 0.7-7% of this variant.^7-9^

According to Bhat, in some cases in which the ulnar artery has a high origin, emerging from the axillary artery, it can even cross the median nerve and supply the brachial biceps muscle.^10^

A superficial ulnar artery has considerable clinical importance. The superficial position, over the flexor muscles, makes it more susceptible to traumas and consequent hemorrhages, which can be serious.^11^ The greatest relevance of knowing about this rare anatomic variant is that the patient can inform the medical and nursing teams, avoiding an increased possibility of unintentional penetration of the superficial ulnar artery during attempts at peripheral venous puncture of the basilic vein in the forearm or of the intermediate cephalic vein during laboratory tests or venous access for infusion of saline and medications.^9^ Pseudoaneurysms in the upper limbs primarily occur in accidents involving penetrating traumas.^12^ Diagnostic investigation methods primarily involve CDU. Magnetic resonance or computed tomography angiography can also be used. Treatment may be surgical or non-surgical. Surgical approaches should be chosen in cases with significant masses, distal ischemia of the limb, or neuropathy. Surgical treatments include arterial ligature, construction of venous bypasses, and partial or total removal of the structure containing the pseudoaneurysm and end-to-end ligature for small pseudoaneurysms. Non-surgical treatment can be achieved by ultrasound-guided administration of thrombin injections.^13^ In the present case, a pseudoaneurysm caused by accidental venous puncture of the superficial ulnar artery clearly demonstrates the importance of acquiring knowledge of variations of arterial anatomy before undertaking any procedure. In such a situation, CDU, which is a widely-available, low-cost, noninvasive method, can serve as the first-choice preoperative examination, or, in cases of suspicion of a superficial ulnar artery, can be used to rule out any possible anatomic variants and avoid complications.
