# Supplementary material for: Superficial ulnar artery pseudoaneurysm
Source: J Vasc Bras. 2023 Dec 4;22:e20230047. doi: 10.1590/1677-5449.202300471 (PMC10706089; doi:10.1590/1677-5449.202300471)
Supplement: Translation

# Translation: Pseudoaneurisma de artéria ulnar superficial

**DOI:** 10.1590/1677-5449.202300472

**Authors:** Mariana Jordão França, Luciana Akemi Takahashi, Graciliano José França, Claudio Augusto Carvalho, Maria Alice Zarate Nissel

**Affiliations:** 1 Universidade Positivo – UP, Curitiba, PR, Brasil.; 2 Universidade Federal do Paraná – UFPR, Hospital de Clínicas, Curitiba, PR, Brasil.

**Keywords:** artéria ulnar, variação anatômica, pseudoaneurisma

## Abstract

A artéria ulnar é o maior ramo terminal da artéria braquial, ela apresenta origem na fossa cubital e é coberta pelos músculos flexores do antebraço. Reportamos uma variação anatômica, na qual a artéria ulnar situava-se em posição superficial no antebraço. Por falta de conhecimento sobre essa variação, ocorreu a lesão após uma tentativa de punção venosa, a qual levou à formação de um pseudoaneurisma.

## INTRODUÇÃO

Normalmente a artéria ulnar apresenta origem na fossa cubital, sendo o maior ramo terminal das duas divisões da artéria braquial. Dirige-se distalmente e medialmente abaixo dos músculos flexores superficiais do antebraço, situando-se sobre o músculo flexor profundo dos dedos. Nos dois terços distais do antebraço, a artéria ulnar situa-se lateralmente ao nervo ulnar no canal de Guyon^1^ . Abaixo do retináculo dos flexores torna-se superficial para formar o arco palmar superficial, junto ao ramo palmar superficial da artéria radial.

No caso da artéria ulnar em trajeto superficial, ela apresenta um percurso não usual, superficial aos músculos flexores do antebraço, podendo originar-se tanto da artéria braquial quanto da artéria axilar. A prevalência da artéria ulnar superficial varia entre 0,7-9,4%^2^ , é maior unilateralmente no braço direito. A ocorrência bilateral tem prevalência 0,01-0,62%^3^ .

Os pseudoaneurismas de artérias periféricas são raros, sendo a frequência muito menor em membros superiores do que em membros inferiores^4^ .

O protocolo foi aprovado pelo Comitê de Ética de nossa instituição (parecer número 5.702.203). O termo de consentimento livre e esclarecido foi assinado para os estudos que envolvem seres humanos.

## PARTE I - SITUAÇÃO CLÍNICA

Paciente do sexo masculino, com 53 anos de idade, mecânico, com histórico de internação prévia para cirurgia coronariana. Procurou avaliação médica no ambulatório de cardiologia devido à formação de uma massa palpável e indolor de aproximadamente 4 cm na face anterior de seu antebraço, depois de uma tentativa de punção venosa durante o procedimento cirúrgico. Após exame físico, foi solicitado eco-Doppler colorido (EDC) arterial dos membros superiores com o objetivo de caracterizar a massa palpável (Figura 1). O exame no membro superior esquerdo revelou uma artéria ulnar superficial pérvia, com origem a partir da artéria axilar (Figura 2), sem estenoses, e a formação de um pseudoaneurisma no seu terço médio, com trombos murais – medindo 2,86 por 1,52 cm (Figuras 3, 4 e 5). Não havia evidência de variação anatômica similar no membro superior direito. Durante o exame de EDC, foi identificada a perviedade da artéria radial e dos arcos palmares superficial e profundo, sendo assim, foi dispensada a realização da manobra de Allen ou similar.

**Figura 1:** Pseudoaneurisma no exame físico.

**Figura 2:** Artéria ulnar superficial com origem na artéria axilar.

**Figura 3:** Eco-Doppler demonstrando a artéria ulnar superficial e o fluxo arterial no saco do pseudoaneurisma.

**Figura 4:** Medida em modo B das dimensões do pseudoaneurisma.

**Figura 5:** Eco-Doppler demonstrando, através do Power Doppler, a artéria ulnar superficial e o fluxo arterial no saco do pseudoaneurisma.

## PARTE II – O QUE FOI FEITO

Após o exame, foi agendada uma consulta no ambulatório de cirurgia vascular, onde foi escolhida cirurgia aberta para tratamento da lesão. O intervalo entre o exame e a cirurgia foi de 50 dias. A cirurgia consistiu numa incisão de 4-5 cm sobre o pseudoaneurisma, controle proximal e distal da artéria ulnar superficial, além da separação das duas veias basílicas (Figura 6). Em seguida, foi realizada a dissecção do pseudoaneurisma, ligando pequenas artérias colaterais. Durante a abertura do pseudoaneurisma, foi observada a trombose total do seu saco do pseudoaneurisma bem como da artéria ulnar distal (Figuras 7 e 8). Por esse motivo, a opção cirúrgica foi a ligadura da artéria ulnar proximal e distal, na qual não houve indício de refluxo e ressecção do pseudoaneurisma trombosado, seguida de revisão da hemostasia e sutura da pele. No pós-operatório imediato, não houve nenhum sinal de isquemia da mão, e o EDC de controle evidenciou um arco palmar superficial e profundo pérvios. O paciente retornou ao ambulatório de cirurgia vascular três meses após a realização da cirurgia (Figura 9), sem queixas, sendo encaminhado para EDC de controle, que revelou ausência de fluxo no trajeto da artéria ulnar superficial (Figura 10), ausência de estenoses na artéria radial e bom fluxo nos arcos palmares (Figuras 11 e 12).

**Figura 6:** Pseudodoaneurisma da artéria ulnar superficial após controle proximal e distal.

**Figura 7:** Pseudoaneurisma após a ressecção.

**Figura 8:** Abertura do pseudoaneurisma demonstrando trombos recentes.

**Figura 9:** Aspecto da cicatriz cirúrgica com três meses de pós-operatório.

**Figura 10:** Ausência de fluxo ao Doppler no trajeto da artéria ulnar superficial.

**Figura 11:** Perviedade do arco palmar superficial ao eco-Doppler colorido.

**Figura 12:** Perviedade do arco palmar profundo ao eco-Doppler colorido.

## DISCUSSÃO

A variação anatômica da artéria ulnar superficial está bem descrita na literatura. Em um estudo de 408 membros superiores, a artéria ulnar superficial foi encontrada em 2,5% dos casos^5^ . Outro estudo com 95 cadáveres concluiu a prevalência da artéria ulnar superficial em 4,2% dos membros, sendo desses 75% no membro superior direito^6^ . Outros autores identificaram que essa variação tem incidência entre 0,7-7%^7,8,9^ .

De acordo com Bhat, em alguns casos de uma origem alta, a partir da artéria axilar, a artéria ulnar pode inclusive cruzar o nervo mediano e suprir o músculo bíceps braquial^10^ .

A artéria ulnar superficial apresenta grande importância clínica. A posição superficial, acima dos músculos flexores, a deixa mais sujeita a traumas e consequentes hemorragias, que podem ser graves^11^ . A maior relevância do conhecimento dessa rara variação anatômica é o paciente poder informar a equipe médica e de enfermagem sobre essa ocorrência, para evitar a grande possibilidade de penetração, de forma não intencional, da artéria ulnar superficial em tentativas de punção venosa periférica da veia basílica no antebraço, ou da veia intermédia do cotovelo em exames laboratoriais, ou acessos venosos para infusão de soro e medicamentos^9^ . Pseudoaneurismas nos membros superiores podem ocorrer principalmente em traumas com acidentes penetrantes^12^ . Métodos de investigação diagnóstica incluem principalmente o EDC. Angiografia por ressonância magnética ou por tomografia computorizada também podem ser utilizadas. O tratamento pode ser cirúrgico e não cirúrgico. Abordagens cirúrgicas devem ser escolhidas em casos de massas significativas, isquemia distal do membro ou neuropatia. O tratamento cirúrgico inclui ligadura arterial, confeção de ponte venosa ou, ainda, a remoção parcial ou total da estrutura contendo o pseudoaneurisma e ligadura término-terminal em pseudoaneurismas pequenos. O tratamento não cirúrgico pode ser feito através injeções de trombina guiadas por ultrassom^13^ . Nesse caso, um pseudoaneurisma, após punção venosa acidental da artéria ulnar superficial, deixa evidente a importância dos conhecimentos das variações anatômicas do percurso arterial antes de qualquer procedimento. Em um cenário como esse, o EDC, por ser um método não invasivo, amplamente disponível e de baixo custo, pode servir como um exame de escolha pré-operatório, ou em casos de suspeita de artéria ulnar superficial, para descartar qualquer possível variação anatômica e evitar complicações.
